# Iron-Catalyzed Chlorination of Titanium Oxides in Molten Salts: A Deep Neural Network-Based Mechanistic Study

**DOI:** 10.3390/ma19091746

**Published:** 2026-04-24

**Authors:** Liangliang Gu, Jie Zhou, Wei Liu, Yuanyuan Chen, Linfei Li, Ronggang Sun, Rong Yu, Xiumin Chen, Yunmin Chen

**Affiliations:** 1 Faculty of Metallurgical and Energy Engineering, Kunming University of Science and Technology, Kunming 650093, China; m17381140041@163.com (L.G.); 15881374909@163.com (J.Z.); xinglongfu63@gmail.com (W.L.); 18768632709@163.com (Y.C.); fff1153708019@outlook.com (L.L.); 19987557941@163.com (R.S.); 13577598050@163.com (R.Y.); 2 National Engineering Research Center of Vacuum Metallurgy, School of Metallurgical and Energy Engineering, Kunming University of Science and Technology, Kunming 650093, China; 3 State Key Laboratory of Complex Nonferrous Metal Resource Clean Utilization, School of Metallurgical and Energy Engineering, Kunming University of Science and Technology, Kunming 650093, China; 4 Beijing DP Technology Co., Ltd., Beijing 100089, China; chenyunmin@dp.tech

**Keywords:** TiO_2_, molten salt chlorination, iron catalysis, AIMD, DeePMD

## Abstract

Molten salt chlorination is a key industrial route for producing titanium tetrachloride (TiCl_4_), yet the atomistic catalytic role of iron (Fe) in the carbothermic chlorination of titanium oxides remains unclear. Here, the chlorination behavior of the NaCl–C–Cl_2_–FeTiO_3_ system was investigated by combining thermodynamic calculations with Ab Initio Molecular Dynamics (AIMD) and Deep Potential Molecular Dynamics (DPMD) simulations. AIMD results show that carbon adjacent to Fe exhibits enhanced reactivity, and that Fe-C synergistic electron transfer promotes both titanium oxide reduction and subsequent titanium chlorination. DPMD results further reveal that Fe not only accelerates these transformations, but also improves interfacial contact among carbon, titanium oxides, and molten salt, thereby enhancing mass transfer and shortening the formation time of TiCl_4_. Temperature-dependent analysis indicates that Fe-C and C-O coordination numbers remain high near 1073 K, where TiCl_4_ formation is efficient and relatively stable. Although increasing temperature can further enhance diffusion, its effect on reaction acceleration is limited, while excessively high temperatures weaken Fe-C interactions and reduce catalytic efficiency. These findings clarify the catalytic mechanism of Fe in molten salt chlorination at the atomic scale and provide theoretical support for process optimization.

## 1. Introduction

Titanium tetrachloride (TiCl_4_) serves as an essential precursor for the production of titanium dioxide [1] and titanium sponge [2], primarily synthesized industrially through fluidized-bed chlorination [3] and molten salt chlorination [4]. For the processing of low-grade vanadium–titanium magnetite or high-titanium slag, molten salt chlorination has emerged as the preferred route for TiCl_4_ production due to its superior mass transfer characteristics [5]. Currently, molten salt chlorination processes are largely focused on ensuring production stability and enhancing yield, leading to a heightened research emphasis on reaction process control. In this context, the catalytic properties of iron have increasingly attracted scientific attention.

It is widely established that the molten salt carbothermic chlorination process involves complex multiphase chemical equilibria; consequently, early research primarily focused on evaluating reaction feasibility and operational parameters through thermodynamic calculations and experimental validation. Ju et al. [6] demonstrated through thermodynamic analysis that the reaction between residual oxygen in the recycled chlorine gas and petroleum coke significantly affects the temperature of the molten salt chlorination furnace, where stable furnace temperature control is crucial for enhancing operational efficiency. Zou et al. [7] calculated the Gibbs free energy for the carbothermic chlorination process under standard states, noting that the complete chlorination of TiO_2_ is achievable at temperatures exceeding 1073 K, and the process remains relatively stable at this point. Li et al. [8] conducted a thermodynamic evaluation and experimental design for high-calcium and magnesium-bearing titanium slag with a TiO_2_ content of 74.6 wt.%, proposing that the stable fluidity of the molten salt system is maintained within the temperature range of 1000~1075 K, and synthesized high-purity TiCl_4_ (g) with a content of 98.8 wt.% from titanium slag in a NaCl-based molten salt system. Furthermore, as a transition metal with strong chemical activity and good electronic conductivity, iron has attracted the attention of several scholars [9]. Deng et al. [10] pointed out that FeCl_3_, formed by the chlorination of iron oxides during the molten salt chlorination process, can exist on the surface of titanium slag via chemisorption; moreover, when FeCl_3_ and carbon coexist, the structure of the titanium slag is effectively dissociated, thereby promoting the reduction and chlorination of titanium oxides. Zhao et al. [11] investigated the equilibrium between FeCl_2_ and FeCl_3_ in molten chlorides and determined the apparent equilibrium constant (K) for the reaction FeCl_2_ + 0.5 Cl_2_ = FeCl_3_. This K value is influenced by the melt composition, chlorine partial pressure, and temperature; these distinct equilibrium relationships determine the various forms of iron in the molten salt, which subsequently affect the coordination structure and ion migration rates within the melt.

While current research on Fe within the molten salt chlorination process remains limited, it has demonstrated significant catalytic promotion in various reduction systems and complex reaction processes [12,13]. Hu et al. [14] investigated the influence of iron powder addition on the carbothermic reduction of chromite for chromium extraction, finding that the addition of iron powder markedly enhances the reduction efficiency while lowering the initiation temperature from approximately 1300 K to around 1150 K; Leuchtenmüller et al. [15] studied the catalytic effects of metal baths on zinc oxide reduction under various experimental conditions, proposing that the addition of iron to the metal bath exerts a potent catalytic effect on the carbothermic reduction of ZnO, with iron-bearing trials proceeding approximately 30 times faster than pure slag trials; Coetsee et al. [16] analyzed the role of metallic iron in the low-temperature carbothermic reduction of manganese oxide using alloy phase analysis, noting that iron not only acts as a solvent to facilitate carbon dissolution into the alloy phase and accelerate the chemical reaction at the carbon–oxide interface but also combines with newly formed manganese to form Fe-Mn-C alloys, which exhibit localized fusion at lower temperatures, thereby increasing the interfacial contact area and achieving a catalytic effect; Negoescu et al. [17] pointed out during the synthesis of nanocomposites from anatase and activated carbon that iron can weaken proximal Ti-O bonds, thus accelerating the formation of oxygen vacancies and facilitating the reaction. In summary, the catalytic effect of Fe is of great significance for reaction promotion; therefore, a profound study of the catalytic mechanism of Fe can provide a theoretical foundation and scientific basis for the optimization of the molten salt chlorination process for titanium oxides.

With the advancement of computational science, molecular dynamics (MD) simulation has increasingly become a robust tool for exploring microstructures and reaction pathways [18,19]. Specifically, ab initio molecular dynamics (AIMD) is capable of elucidating atomic interaction mechanisms, characterizing interatomic chemical bonds [20], and uncovering the interfacial behavior of molten salts at elevated temperatures [21]. Meanwhile, deep potential molecular dynamics (DPMD) facilitates the efficient simulation of structural evolution and intermolecular interactions [22], prompting extensive research utilizing MD simulations [23,24,25]. Stefanis et al. [26] employed AIMD to calculate the thermophysical and transport properties of the MgCl_2_–NaCl–KCl ternary system, confirming the reliability of the simulation through comparisons with experimental and theoretical benchmarks. Hazebroucq et al. [27] analyzed the coordination numbers and self-diffusion coefficients of KCl and NaCl based on density functional theory (DFT), showing that the MD results align well with experimental observations. Guo et al. [28] used MD simulations to study the liquid-phase physical properties of chlorination slag after adding Na_2_SiO_3_, noting that the additive reduces the density and viscosity of the mixed melt—a finding supported by experimental data. He et al. [29] investigated the influence of different additives on the structure and properties of molten salt systems at 1200 K and 1.01 MPa via MD simulations, discovering that Fe addition enhances Al^3+^−Al^3+^ interactions. However, in the study of molten salt chlorination, analyzing microstructural changes and reaction mechanisms through experimental methods often faces substantial limitations; furthermore, conventional characterization techniques struggle to capture transient intermediates in high-temperature molten salts. Consequently, AIMD and DPMD have emerged as effective methodologies for probing the structural properties [30,31], transport characteristics [32,33,34], and reaction mechanisms [35,36] of molten salt systems. Deng et al. [37] determined parameters such as radial distribution functions, coordination numbers, and diffusion coefficients through MD simulations, noting that an increasing concentration of CaCl_2_ allows Ca^2+^ ions to complex with more Cl^−^ ions to form ionic clusters, thereby reducing the diffusion coefficients of Na^+^, Mg^2+^, Ca^2+^, and Cl^−^. Based on MD simulations, Xu et al. [38] analyzed the mean squared displacement (MSD) and radial distribution function (RDF) of the NaCl–MgCl_2_–CaCl_2_–FeCl_2_/FeCl_3_ system, pointing out that Fe^2+^ and Fe^3+^ ions form stronger bonds with Cl^−^ than Na^+^ or Ca^2+^, which promotes the formation of longer-chain structures and larger ionic clusters. Furthermore, Zhang et al. [39] combined AIMD and DPMD to investigate the reaction mechanism of the carbothermic chlorination of titanium oxides, revealing the conversion mechanism from TiO_2_ to TiCl_4_ and elucidating the chemical species and transformation pathways of chlorine in the system; ultimately, experimental analysis validated that NaCl can act as a chlorine source participating in the chlorination of titanium oxides.

Although research on molten salt systems has been extensive, reports on the behavioral laws and catalytic mechanisms of Fe during the molten salt chlorination of titanium oxides remain scarce; in particular, the catalytic mechanism at the atomic scale has yet to be revealed. To this end, ab initio molecular dynamics (AIMD) and deep potential molecular dynamics (DPMD) simulations were introduced in this study. By analyzing the electronic structures, inter-component interactions, and transport properties of the NaCl–C–Cl_2_–FeTiO_3_ system, the catalytic role of Fe during the reaction process was discussed. Furthermore, the formation process of titanium chlorides and the associated reaction mechanisms under the influence of iron were elucidated.

## 2. Method

### 2.1. Thermodynamic Calculations

Thermodynamic calculations in this study were based on the principle of Gibbs free energy minimization. The equilibrium compositions of the NaCl–C–Cl_2_–FeTiO_3_ system were calculated using Factsage 8.0 software [40]. The input parameters were configured as follows: NaCl (5000 kmol), C (1200 kmol), Cl_2_ (2000 kmol), and FeTiO_3_ (350 kmol). The pressure was set at standard atmospheric pressure (1 atm), with the temperature range spanning from 273 to 1473 K. Additionally, the amounts of each equilibrium component at 1073 K were obtained.

### 2.2. Kinetic Calculations

#### 2.2.1. AIMD Methods and Parameters

In this study, the structural model of the molten salt chlorination reaction system was constructed using Materials Studio 2017 software [41]. Periodic boundary conditions were applied to maintain a constant number of atoms and eliminate boundary effects. The as-constructed NaCl–C–Cl_2_–FeTiO_3_ system consisted of 527 atoms in total, including 127 Na, 172 Cl, 92 C, 54 Ti, 78 O, and 4 Fe atoms. The supercell parameters were set as A = B = 22.480 Å, C = 24.050 Å, and α = β = γ = 90°. Subsequently, geometry optimization of the constructed model was performed using the Cambridge Sequential Total Energy Package (CASTEP) module [42] within Materials Studio.

**Figure 1:**
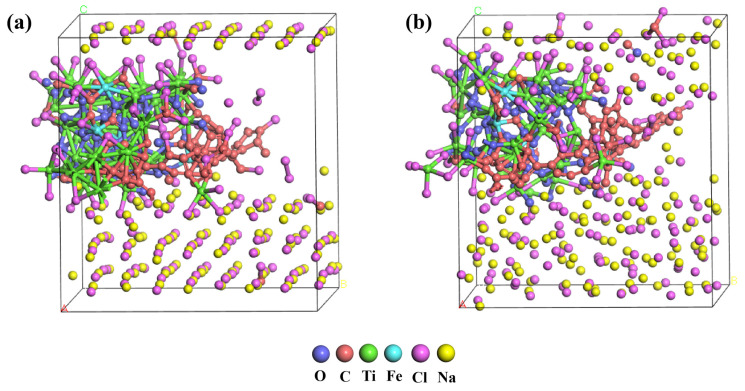
(**a**) Structure after geometric optimization. (**b**) Structure after molecular dynamics simulation.

Subsequently, molecular dynamics simulations were carried out on the optimized structures using the Vienna Ab initio Simulation Package (VASP 5.4.4) [43]. The cutoff energy was set to 600 eV. The simulations were performed in the NVT ensemble using a Nosé–Hoover thermostat [44]. The convergence criteria for the self-consistent field (SCF) and atomic relaxation were set to 1 × 10^−5^ eV and 1 × 10^−4^ eV, respectively. A total of 5000 steps were executed with a time step of 0.5 fs. The system temperatures were set at 1073 K, 1173 K, 1273 K, 1373 K, and 1473 K, and the total simulation time was 2.5 ps. The structure after geometric optimization and simulation is shown in Figure 1.

To further investigate the electronic structural properties and inter-component interactions of the system, the CASTEP module within the Materials Studio software package was employed to calculate electronic properties, such as bond populations, lengths, and Mulliken charges, for specific frames extracted from the molecular dynamics simulations. The Perdew–Burke–Ernzerhof (PBE) exchange-correlation functional based on the generalized gradient approximation (GGA) [45] was adopted. The calculations were performed with a k-point grid of 1 × 1 × 1 and an energy cutoff of 517 eV. The tolerances for maximum displacement, maximum force, and energy were set at 0.002 Å, 0.05 eV/Å, and 2.0 × 10^−6^ eV/atom, respectively.

#### 2.2.2. DeePMD Model Construction and Parameter Setting

**Figure 2:**
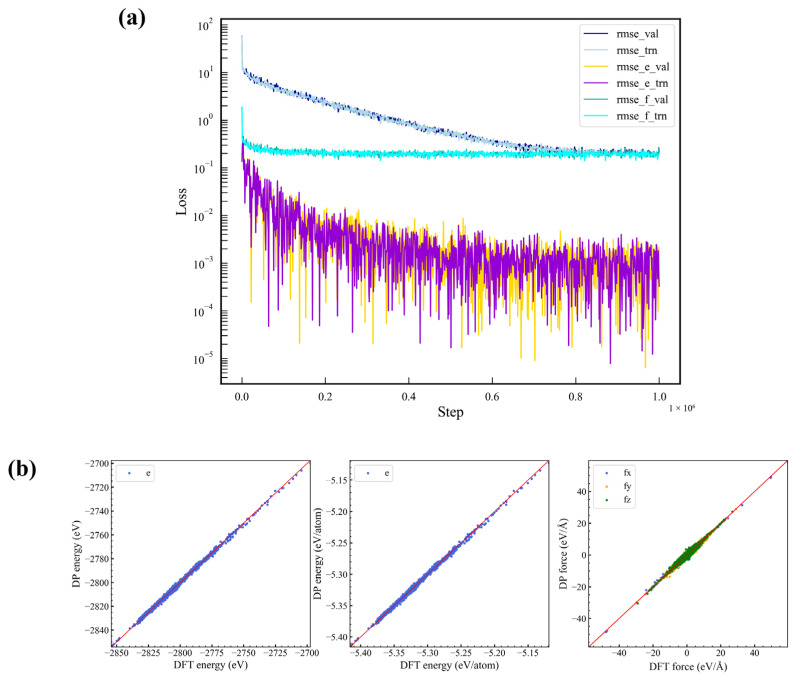
(**a**) Fitting curves of energy and force, (**b**) scatter plots of the predicted values (DeePMD) and the actual values (DFT) of the validation set.

DeePMD [46] is a neural network-based deep simulation method that enables large-scale molecular dynamics simulations by training potential energy functions using data obtained from ab initio molecular dynamics (AIMD). In this study, based on a short-bond evaluation method [47], data were collected from five sets of AIMD simulations using DeepMD-kit [48] to train the dataset and obtain high-dimensional potential functions. Specifically, 90% of the sample data were randomly selected as the training set, while the remaining 10% served as the validation set. The descriptor keyword “type” was set to “se_a”, with “rcut_smth” at the default value and the cutoff radius set to 8 Å. The nodes for the embedding neural network used to generate descriptors were set at (20, 40, 80), and the nodes for the deep neural network used to train the potential function model were (200, 200, 200). The initial weight coefficients were set at $p_{e}^{\mathrm{start}}$ = 0.02 and $p_{f}^{\mathrm{start}}$ = 1000, while the final weight coefficients were $p_{e}^{\mathrm{limit}}$ = 1 and $p_{f}^{\mathrm{limit}}$ = 1. The number of training steps was specified as 1 × 10^6^. The resulting loss curves, used to examine the convergence trends of energy and force, are shown in Figure 2a. It can be observed that the training set results are essentially consistent with those of the validation set, indicating a high-quality fit and confirming the model’s suitability for subsequent simulation tasks. Figure 2b displays a scatter plot of the predicted values (DP) versus the true values (DFT) for the validation set, which evaluates the model’s fitting capability. In the validation results, the root-mean-square error (RMSE) for the energy of each atom is 1.525 × 10^−3^ eV, and the RMSE for the forces across the three coordinate directions is approximately 10^−1^ eV/Å.

Based on the training results, the structural model for the NaCl–C–Cl_2_–FeTiO_3_ system was constructed and imported into the LAMMPS software (2 August 2023 version) [49]. This system comprised a total of 10,973 atoms, including 4499 Na, 4586 Cl, 766 C, 710 O, 349 Ti, and 63 Fe atoms. The time step was set to 0.5 fs, with a total simulation time of 10 ns. The supercell parameters were specified as a = b = 59.609 Å, c = 253.39 Å, and α = β = γ = 90°, with a minimum iteration of 5000 steps. The simulation temperatures were set at 873 K, 973 K, 1073 K, 1173 K, 1273 K, and 1373 K. Additionally, a NaCl–C–Cl_2_–TiO_2_ system was constructed as a control, containing 10,910 atoms (4499 Na, 4586 Cl, 766 C, 710 O, and 349 Ti). The calculation for the control system was performed at 1073 K, with all other parameters remaining consistent with those of the NaCl–C–Cl_2_–FeTiO_3_ system.

## 3. Results and Discussion

### 3.1. Thermodynamic Calculation Results

**Figure 3:**
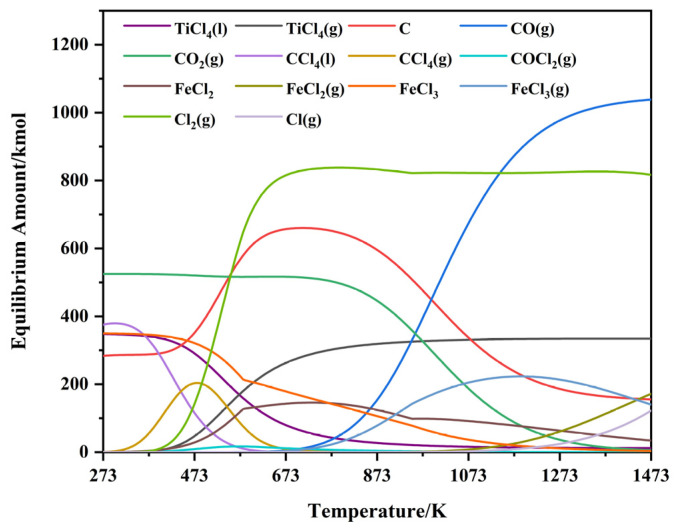
Equilibrium composition diagram of the NaCl–C–Cl_2_–FeTiO_3_ system.

The equilibrium compositions of the NaCl–C–Cl_2_–FeTiO_3_ system calculated via FactSage 8.0 across a temperature range of 273~1473 K are illustrated in Figure 3. The figure identifies the primary products and certain high-content intermediates formed within this temperature interval. As illustrated in Figure 3, the target product TiCl_4_ exists in the liquid phase at room temperature and begins to transition into the gas phase upon reaching its boiling point at approximately 400 K. As the temperature rises to 1073 K, the TiCl_4_(g) content reaches 330.9 kmol and subsequently tends to stabilize. Iron chlorides exist predominantly as solid FeCl_3_ at low temperatures (273~373 K); its content decreases with rising temperature, reaching the melting point at around 573 K to form liquid FeCl_3_, and eventually drops to 3.46 kmol at 1473 K. Previous research by Bonsack et al. [50] demonstrated that FeCl_3_ is prone to dechlorination and reduction to FeCl_2_ under reducing environments and limited chlorine potential. Consequently, the figure reveals the conversion of FeCl_3_ to FeCl_2_ starting at approximately 373 K. At this stage, FeCl_2_ is in the solid phase, reaching a peak content of 143 kmol at approximately 773 K, before gradually decreasing and reaching its melting point at around 943 K to transition into the liquid phase, eventually declining to 34 kmol at 1473 K. Simultaneously, FeCl_3_ begins generating gaseous FeCl_3_(g) upon reaching its boiling point at approximately 623 K, peaking at 221 kmol at 1223 K, and subsequently decreasing to 140 kmol at 1473 K as the temperature continues to rise. In addition, when the system temperature is below 673 K, the reaction between C and Cl_2_(g) mainly produces CCl_4_(l) and CCl_4_(g). As the temperature further increases above 773 K, the contents of both CCl_4_(l) and CCl_4_(g) decrease to below 1 kmol. Beyond 773 K, the C content gradually decreases alongside the CO_2_(g) content due to the Boudouard reaction. In the high-temperature region above 973 K, carbon oxides exist predominantly as CO(g). COCl_2_(g) reaches a peak content of 17.09 kmol at around 573 K but, owing to its susceptibility to decomposition at high temperatures, its content gradually decreases to approximately 1 kmol by 1073 K.

**Figure 4:**
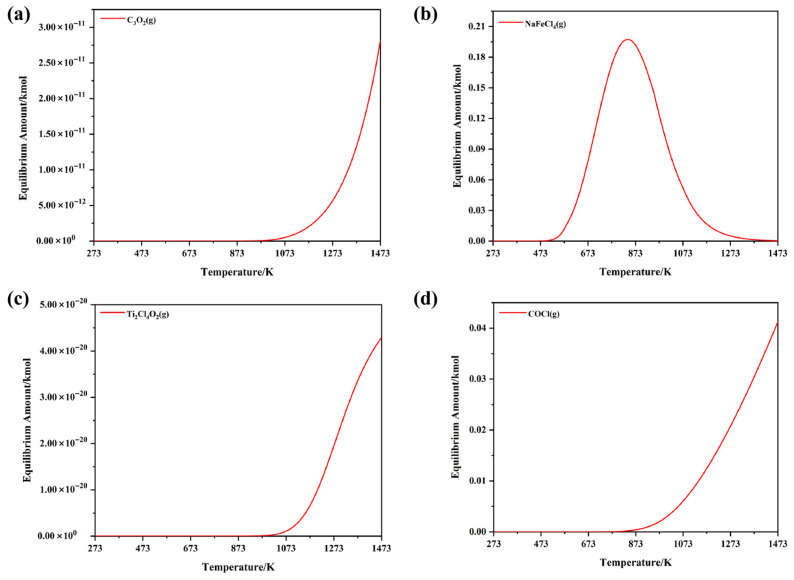
Partial composition diagram of intermediate products in the NaCl–C–Cl_2_–FeTiO_3_ system: (**a**) Variation in the content of C_3_O_2_(g) with increasing temperature; (**b**) Variation in the content of NaFeCl_4_(g) with increasing temperature; (**c**) Variation in the content of Ti_2_Cl_4_O_2_(g) with increasing temperature; (**d**) Variation in the content of COCl(g) with increasing temperature.

In addition to the aforementioned products, specific characteristic components within the NaCl–C–Cl_2_–FeTiO_3_ system were analyzed and plotted separately in Figure 4. The results indicate the presence of species such as C_3_O_2_(g), NaFeCl_4_(g), Ti_2_Cl_4_O_2_(g), and COCl(g) within specific temperature ranges. Figure 4a shows that C_3_O_2_(g) gradually forms in the NaCl–C–Cl_2_–FeTiO_3_ system between 1023 and 1473 K. The C_x_O_y_ structure suggests it may serve as an intermediate state for carbon oxides during the reduction process, Geiger [51] noted that when CO(g) and CO_2_(g) coexist under low oxygen potential, C−O−C chain species can form locally, leading to the generation of C_3_O_2_(g). As shown in Figure 4b, NaFeCl_4_(g) begins to form after 523 K, reaching a peak content at approximately 873 K before gradually decreasing, with a stable temperature range of 523~1373 K. This compound is formed via Na−Fe−Cl coordination, suggesting a potential interaction between Na and Fe within the system. Bergeron [52] previously reported that NaFeCl_4_ can form from NaCl and FeCl_3_ in high-temperature chlorination environments, acting as an effective chlorinating agent to transfer chlorine. Ti_2_Cl_4_O_2_(g), shown in Figure 4c, begins to form above 1023 K and exists within the 1023~1473 K range, characterized by Ti−O−Cl coordination. Dunn et al. [53] pointed out that an equilibrium between TiO_2_ and TiCl_4_ can be established on rutile surfaces to produce gaseous titanium oxychlorides. Considering that West et al. [54] identified Ti_2_Cl_4_O_2_ as a dimer of TiOCl_2_, this species likely represents an unstable transition intermediate during the conversion of TiO_2_ to TiCl_4_(g). Figure 4d reveals that COCl(g) starts forming after 873 K, with its content increasing alongside the temperature across the 873~1473 K range. Rossi et al. [55] noted that COCl can be generated through the collision and binding of CO and Cl; furthermore, when adsorbed Cl atoms are abundant, COCl can further react with Cl to produce COCl_2_. Combined with Amama et al.’s [56] finding that phosgene (COCl_2_) can further convert into C_2_O_2_Cl_2_ under certain conditions, these results suggest that carbon–oxygen-chlorine compounds exist in various coordination forms. However, due to their low concentrations, they are considered only as transient intermediates participating in the reaction.

As indicated in Figure 3, the content of the target product TiCl_4_(g) tends to stabilize above 1073 K. Consequently, this study focuses on the reaction mechanism of the NaCl–C–Cl_2_–FeTiO_3_ system at this specific temperature. The equilibrium components and their corresponding contents at 1073 K are summarized in Table 1. As presented in Table 1, following the carbothermic chlorination of ilmenite (FeTiO_3_), the Ti-based species consist primarily of TiCl_4_(g), accompanied by minor amounts of titanium oxides (TiO), low-valence titanium chlorides (TiCl(g)), titanium oxychlorides (TiClO(g)), and TiC. Regarding the Fe-based species, in addition to FeCl_2_, FeCl_2_(g), FeCl_3_, and FeCl_3_(g), small quantities of iron oxides (FeO), low-valence iron chlorides (FeCl(g)), iron oxychlorides (FeOCl(g)), iron carbides (Fe_2_C), and FeTi. Furthermore, the reaction of carbon with ilmenite and Cl_2_ yields CO(g), CO_2_(g), COCl_2_(g), and COCl(g), along with the formation of trace amounts of low-valence carbon chlorides CCl(g).

### 3.2. AIMD Results

**Figure 5:**
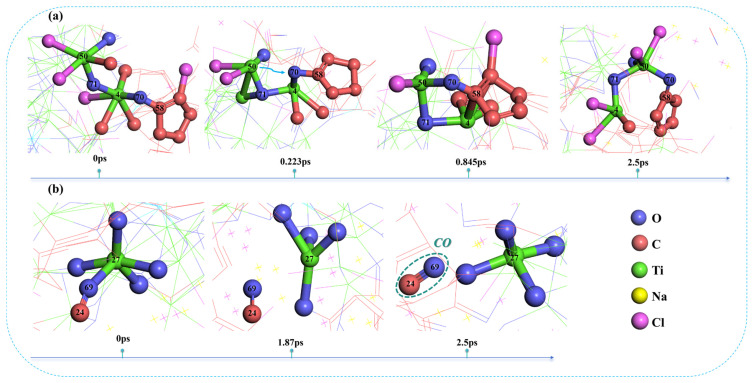
Structural snapshots of the reduction process of titanium oxides without Fe participation: (**a**) First structure; (**b**) Second structure.

**Figure 6:**
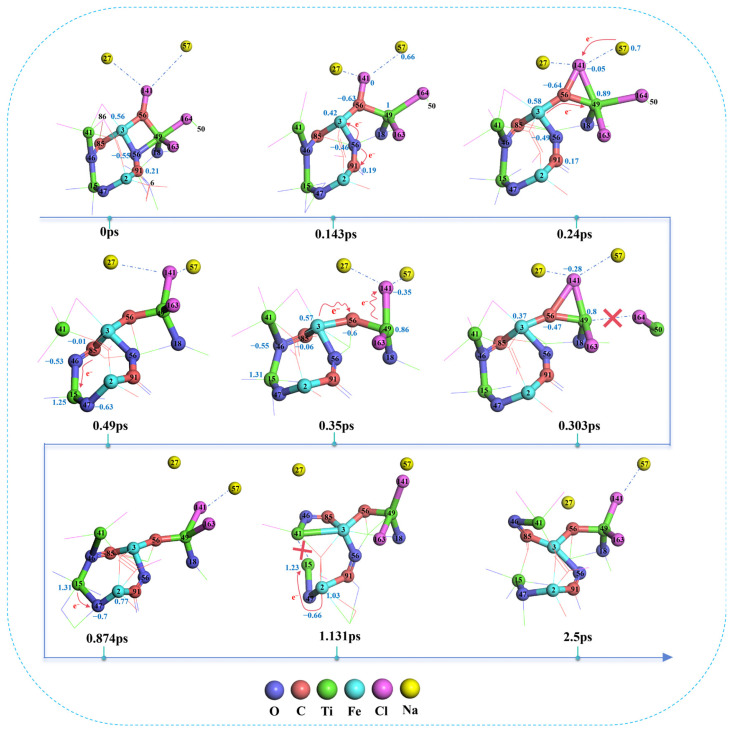
Structural snapshots of the Fe-catalyzed reduction process of titanium oxides.

**Figure 7:**
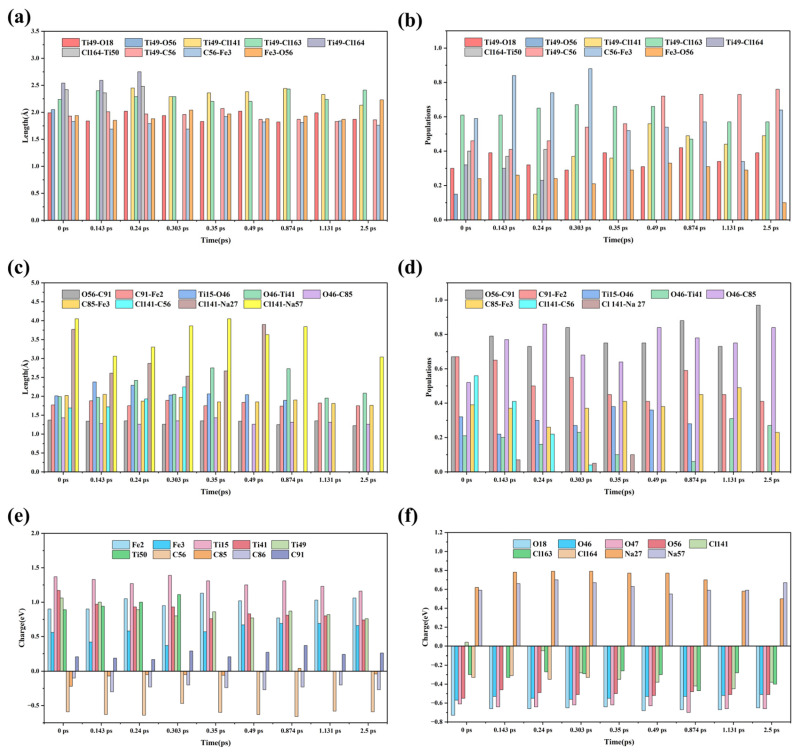
Temporal evolution of selected bond lengths, bond populations, and charges in AIMD simulations: (**a**,**c**) bond lengths as a function of time; (**b**,**d**) bond populations as a function of time; (**e**,**f**) charges as a function of time.

To further investigate the interactions between components during the production of TiCl_4_(g) via molten salt chlorination, ab initio molecular dynamics (AIMD) simulations were performed on the NaCl–C–Cl_2_–FeTiO_3_ system at 1073 K. The structural evolution and electronic properties of titanium oxides within the simulation results were analyzed, as illustrated in Figure 5, Figure 6 and Figure 7. (Detailed data are provided in the Supplementary Materials).

As shown in Figure 5a, at 0 ps, C58 in the carbon ring structure is bridged to Ti4 through O70, while Ti4 is further bridged to Ti50 through O71. With the migration of the carbon ring, the distance between O70 and Ti50 gradually decreases. By 0.223 ps, an O70−Ti50 bond is formed with a bond length of 2.69 Å, whereas the O70−Ti4 bond length increases from 1.9 Å at 0 ps to 1.95 Å, indicating that their interaction is gradually weakening. At 0.845 ps, the O70−Ti50 bond length further decreases from 2.69 Å to 1.97 Å, suggesting that the bond tends to stabilize, while the distance between O70 and Ti4 reaches 3.15 Å, indicating that bond cleavage has occurred. This demonstrates that O70 has been reduced and extracted from the titanium oxide structure by C58 at this stage. As shown in Figure 5b, at 0 ps, C24 is bridged to Ti27 through O69, and the bond lengths of C24−O69 and O69−Ti27 are 1.17 Å and 2.23 Å, respectively. By 1.87 ps, C24 directly reduces and extracts O69 from Ti27. Meanwhile, the C24–O69 bond length decreases from 1.17 Å to 1.14 Å, indicating that the resulting carbon oxide has formed a stable CO structure.

As illustrated in Figure 6 and Figure 7a,c, at 0 ps, Fe3 is bridged to Ti49 via O56, with Fe3−O56 and O56−Ti49 bond lengths of 1.94 Å and 2.05 Å, respectively. O56 is further bridged to Fe2 through C91, with the O56−C91 and C91−Fe2 bond lengths measured at 1.37 Å and 1.77 Å. Meanwhile, Fe2 is bridged to Ti15 via O47, characterized by Fe2−O47 and O47−Ti15 bond lengths of 1.87 Å and 1.75 Å, respectively. Ti15 is also bridged to C85 through O46, with Ti15−O46 and O46−C85 bond lengths of 2.00 Å and 1.43 Å, whereas C85 is directly bonded to Fe3 with a bond length of 2.02 Å. In addition, Ti49 within the structure is directly bonded to O18, Cl163, and Cl164, with respective bond lengths of 1.99 Å (Ti49−O18), 2.24 Å (Ti49−Cl163), and 2.54 Å (Ti49−Cl164). Apart from O56, Fe3 and Ti49 are also bridged by C56, with Fe3−C56 and C56−Ti49 bond lengths of 1.83 Å and 1.93 Å, respectively. Concurrently, C56 is connected to Cl141 (1.69 Å), and O46 is bonded to Ti41 (1.99 Å). In addition, Na27 and Na57 in molten NaCl are located 3.77 Å and 4.15 Å away from Cl141, respectively.

As shown in Figure 7e,f, both Fe2 and Fe3 are positively charged, with values of 0.9 and 0.56, respectively. Ti15, Ti41, and Ti49 also carry positive charges of 1.37, 1.17, and 1.06, respectively. Conversely, O18, O46, O47, and O56 are all negatively charged, with values of −0.73, −0.57, −0.61, and −0.55, respectively. Cl141, which originates from the dissociated chlorine molecule, carries a slight positive charge of 0.04, whereas Cl 163 and Cl164 are both negatively charged (−0.3 and −0.33, respectively). Notably, the carbon atoms within the structure exhibit both positive and negative charges: C91 has a charge of 0.21, while C85 and C56 carry charges of −0.22 and −0.59, respectively. This differentiation is closely related to the distinct local atomic environments of the three carbon atoms. Specifically, C91 is bonded to Fe2, O56, and O6. Since oxygen possesses a significantly higher electronegativity than carbon, and Fe2—which is concurrently bonded to O47—exists in an electron-deficient state, C91 exhibits positive charging characteristics. C85 is bonded to O46, Fe3, and C86; although O46 attracts a portion of the electron density, Fe3 carries a significantly higher positive charge than Fe2, leading to a charge of −0.22 on C85. Meanwhile, C56 is bonded to Fe3, Ti49, and Cl 141 simultaneously, where Cl 141 originates from a dissociated Cl2 molecule and is essentially electrically neutral. This leads to electron enrichment around C56, Moreover, because there is no atom in the bonded structure capable of accepting electrons from C56, the electrons become polarized toward C56, causing its charge to reach −0.59.

At 0.143 ps of the simulation, O56 transfers electrons to the adjacent positively charged Fe3 and C91, causing its own charge to shift positively from −0.55 to −0.46. During this process, the Fe3−O56 and O56−C91 bond lengths shorten from 1.94 Å and 1.37 Å to 1.85 Å and 1.34 Å, respectively, leading to a progressive strengthening of these interactions. Subsequently, under the traction of C91, the O56−Ti49 distance increases from 2.05 Å to 2.84 Å, resulting in bond cleavage; this signifies the detachment of O56 from the titanium oxide structure through the synergistic effect of Fe and C. By 0.24 ps, Fe3 transfers electrons to Ti49 via the C56 bridge, shifting the Ti49 charge negatively from 1.00 to 0.89. Concurrently, Na57 transfers electrons to Cl141, shifting its charge from 0 to −0.05. During this stage, the distances between Cl141 and its neighboring Na27, Na57, and C56 atoms increase to 2.87 Å, 3.30 Å, and 1.93 Å, respectively. The weakening of these interactions facilitates the initial bonding between Cl141 and Ti49.

At 0.303 ps, Cl164 accepted the electrons provided by Ti50 and then transferred them to Ti49, causing its charge to shift negatively from 0.89 to 0.8. During the process, the bond length of the Ti50−Cl164 bond decreased from 2.48 Å to 2.33 Å, and the population increased from 0.41 to 0.47, as shown in Figure 7b,d. The interaction strengthened and tended toward stable bonding, while the interaction between Ti49 and Cl164 gradually weakened, with the distance increasing to 3.18 Å and bond cleavage occurring. When the simulation reached 0.35 ps, the population of the Ti49–Cl141 bond reached 0.36, and the bonding tended to stabilize. Meanwhile, C56 accepted the electrons transferred from Fe3, causing its charge to shift negatively from −0.47 to −0.6, and Cl141 accepted the electrons transferred from Ti49, causing its charge to shift negatively from −0.28 to −0.35. During the process, the C56−Cl141 bond gradually weakened, and the distance increased from 2.25 Å to 2.48 Å, leading to the cleavage of the carbon–chlorine bond. By 0.49 ps, C85 transferred electrons to Ti15 via the bridging O46, causing its charge to shift negatively from 1.31 to 1.25. During this period, the bond lengths of C85−O46 and O46−Ti15 shortened to 1.26 Å and 2.04 Å, respectively, and the interaction gradually strengthened. Under the action of C85 on O46, the distance of the O46−Ti41 bond increased from 2.75 Å to 3.24 Å, resulting in bond cleavage. By 0.874 ps of the simulation, Ti15 transferred electrons to O47, causing the charge to shift positively from 1.25 to 1.31, while O46 maintained its charge of −0.53. During the process, the population of Ti15−O46 decreased from 0.36 to 0.28. It can be seen that while the Ti15–O46 bond weakened, Ti41 began to re-bond with O46. Subsequently, by 1.131 ps, Fe2 transferred electrons to Ti15 via the bridging oxygen O47, causing its charge to shift negatively from 1.31 to 1.23. During this period, the bond length of O47−Ti15 shortened from 2.33 Å to 2.11 Å. Under the traction of O47, the interaction between Ti15 and O46 gradually weakened, ultimately resulting in the cleavage of the titanium–oxygen bond.

In summary, it can be seen that the carbon adjacent to iron in the system has high reactivity. During the reduction of titanium oxides, the activated carbon can either directly acquire electrons from oxygen or transfer electrons to Ti through the bridging oxygen, weakening the Ti-O bond and promoting the formation of C-O bonds, thus leading to the cleavage of titanium–oxygen bonds. During the chlorination stage, Fe can directly transfer electrons to C or transfer electrons to Ti through the bridged carbon, facilitating the cleavage of carbon–chlorine bonds and the formation of titanium–chlorine bonds, thereby promoting the transfer of chlorine from carbon to titanium. The reduction and chlorination behavior of titanium described above essentially occurs as a result of the cooperative action between iron and carbon in the reduction and chlorination process of titanium oxides.

Furthermore, comparing the reduction process of titanium oxides without Fe involvement in Figure 5 with the structures involving Fe in Figure 6, it can be observed that, in the absence of iron, the titanium–oxygen bond dissociation is only observed after the simulation reaches 1.87 ps. However, in the presence of iron, the titanium–oxygen bonds are dissociated after the simulation reaches 0.143 ps and 0.49 ps, respectively. This indicates that Fe plays a catalytic role in promoting the reduction process of titanium oxides.

### 3.3. DPMD Results

Due to the limitations in both time and spatial scales of ab initio molecular dynamics simulations, deep potential molecular dynamics simulations were further conducted to better elucidate the catalytic mechanism of Fe in the NaCl–C–Cl_2_–FeTiO_3_ system.

#### 3.3.1. Analysis of the Catalytic Mechanism of Fe

**Figure 8:**
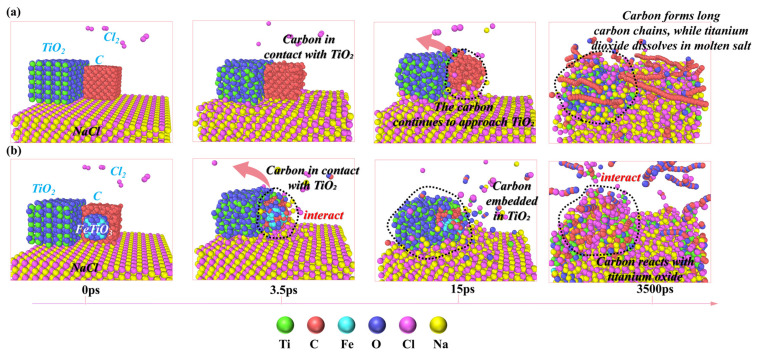
Component behavior during the molten salt carbothermic chlorination process of titanium oxides: (**a**) NaCl–C–Cl_2_–TiO_2_ system; (**b**) NaCl–C–Cl_2_–FeTiO_3_ system.

Figure 8 presents a comparison between the NaCl–C–Cl_2_–TiO_2_ system and the NaCl–C–Cl_2_–FeTiO_3_ system under the molten salt carbothermic chlorination process at 1073 K, aiming to further understand the influence and mechanism of iron on the carbothermic chlorination reaction process. As shown in Figure 8a, at 3.5 ps, carbon in the NaCl–C–Cl_2_–TiO_2_ system begins to make contact with titanium oxide, but no interaction occurs between them at this point. In contrast, at the same time, in Figure 8b, carbon clusters in the NaCl–C–Cl_2_–FeTiO_3_ system interact significantly with titanium oxide under the influence of iron oxide, and gradually embed into it. The figure clearly shows that Fe-C and Ti-C bonds have formed, which corresponds to the previous ab initio molecular dynamics simulation results (Figure 6) and is consistent with the conclusions from the thermodynamic calculations. By 15 ps, as shown in Figure 8a, although carbon in the iron-free system begins to gradually embed into titanium oxide, it still maintains its cluster form and does not react with the metal oxide. However, in Figure 8b, under the influence of partially reduced iron, the interaction between carbon clusters and titanium oxide continues to strengthen, and the reduction of some titanium and iron oxides accompanies the embedding of carbon into TiO_2_. During the subsequent simulation process, a significant impact of iron on the behavior of carbon can be observed. By the time the simulation reaches 3500 ps, as shown in Figure 8a, carbon in the NaCl–C–Cl_2_–TiO_2_ system is primarily adsorbed on the molten salt surface in the form of long carbon chains. At this point, some reduced and chlorinated titanium oxides exhibit better affinity for sodium chloride, essentially completely immersed in the NaCl molten salt, preventing effective contact between titanium oxide and carbon, resulting in a low degree of titanium oxide reduction. In Figure 8b, with the presence of iron in the NaCl–C–Cl_2_–TiO_2_ system, carbon undergoes significant decomposition, and the decomposed carbon exhibits high activity, rapidly combining with oxygen from the metal oxide and gradually forming CO and CO_2_. Interestingly, the CO and CO_2_ in the system tend to form chain-like structures in the form of C_n_O_n+1_, and titanium predominantly exists as low-valence titanium oxide and low-valence titanium chloride.

**Figure 9:**
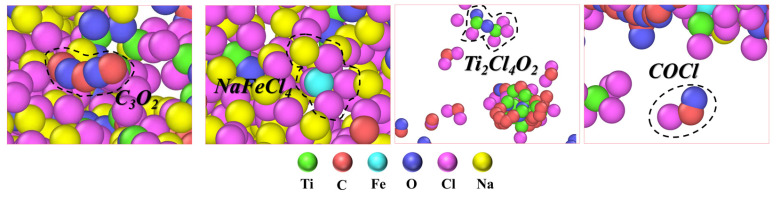
Partial intermediate products in the molten salt carbothermic chlorination process of the NaCl–C–Cl_2_–FeTiO_3_ system.

From the above comparison, it is evident that in the molten salt carbothermic chlorination process for producing TiCl_4_, without the involvement of iron, the interaction between titanium dioxide and carbon is weak and their contact is difficult, resulting in a slower reaction rate. However, in the presence of iron, as analyzed in the previous AIMD results, iron can effectively facilitate electron transfer between titanium dioxide and carbon, thereby promoting the dissociation and activation of carbon, significantly accelerating the reduction and chlorination reactions. Additionally, during the DPMD simulations, intermediate products such as C_3_O_2_, NaFeCl_4_, Ti_2_Cl_4_O_2_, and COCl were found to form, as shown in Figure 9. Based on the previous thermodynamic calculations and related literature [52,55], it is known that these intermediate products can form under specific conditions and, as the system environment changes, can further transform into other products to promote the progress of the reaction. Specifically, C_3_O_2_, as a transient carbon–oxygen compound, may decompose into species such as CO or CO_2_ and continue to participate in subsequent reactions; NaFeCl_4_ is formed through the complexation of NaCl and FeCl_3_ in the molten salt, and its formation indicates a certain interaction between Na and Fe, while such complexation may also suppress the volatilization of FeCl_3_; the identification of Ti_2_Cl_4_O_2_ further reflects the reliability of the simulation, as this species is commonly regarded as an intermediate in the transformation from TiO_2_ to TiCl_4_; COCl, as an intermediate formed by the combination of Cl radicals with CO, contains both C and Cl in its structure and thus exhibits both reducing and chlorinating characteristics. The identification of these intermediate species not only verifies the consistency between the simulation results and thermodynamic predictions, but also directly reflects the complex multiphase characteristics of the molten salt carbothermal chlorination process.

**Figure 10:**
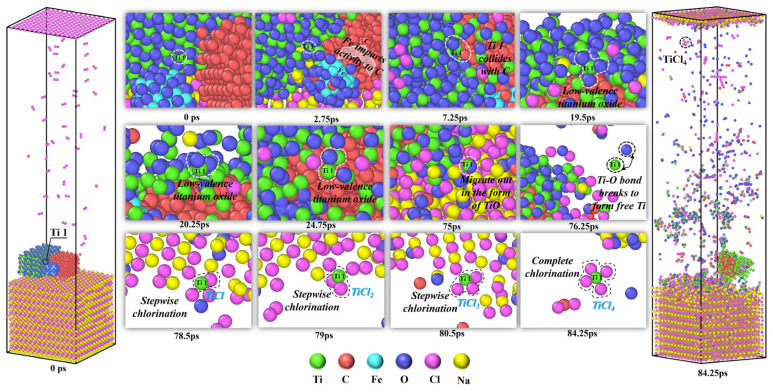
Catalytic effect of Fe on the molten salt carbothermic chlorination process of titanium oxides.

Although previous studies have reported on the chlorination reaction mechanism of the NaCl–C–Cl_2_–TiO_2_ system [39], these studies did not focus on the effect of the interaction between carbon and titanium dioxide on the reduction process. Therefore, to further illustrate the catalytic role of iron in the molten salt chlorination process of titanium oxides, the formation process of TiCl_4_ in the NaCl–C–Cl_2_–FeTiO_3_ system is analyzed, as shown in Figure 10.

As shown in Figure 10, at 0 ps, Ti1 is located on the surface of the titanium oxide above the molten salt, with six oxygens coordinating with Ti1. As the simulation progresses, iron oxide migrates towards the carbon clusters, and some Fe is reduced by carbon. At 2.75 ps, carbon, with high reactivity after contacting Fe, gradually embeds into the titanium oxide and begins to react with it. At 7.25 ps, a reduction trend is observed for the Ti1 near carbon. From 7.25 ps to 19.5 ps, under the influence of the reduced metallic Fe, the interaction between carbon and titanium oxide continues to strengthen. Subsequently, the oxygen atoms bonded to Ti1 are gradually reduced and detached by carbon. From 19.5 ps, the titanium oxide centered around Ti1 begins to diffuse and wrap around the surface of the carbon cluster, with the number of oxygens coordinating with Ti1 decreasing from six to three. As the simulation continues, the titanium oxide structure centered around Ti1 migrates along the surface of the carbon cluster, while the oxygen atoms on Ti1 continue to detach from the titanium oxide structure under the reducing atmosphere created by carbon. At 20.25 ps, the number of oxygens bonded to Ti1 decreases further to two. With continuous reduction of titanium oxide by carbon, at 24.75 ps, Ti1, now bonded to only one oxygen, contacts an adjacent titanium and forms Ti_2_O, but it dissociates into TiO. Subsequently, the TiO centered around Ti1 continues to migrate irregularly on the surface of the carbon cluster. By 75 ps, TiO, centered around Ti1, detaches from the molten salt surface. At 76.25 ps, the free TiO molecules dissociate, and the resulting Ti1 moves rapidly within the system. By 78.5 ps, Ti1 reaches the molten salt surface and bonds with Cl in NaCl to form TiCl. Subsequently, at 79 ps and 80.5 ps, Ti1 sequentially bonds with Cl in the molten salt to form TiCl_2_ and TiCl_3_. By 84.25 ps, after bonding with the fourth Cl in the molten salt, Ti1 forms TiCl_4_, which escapes from the molten salt, completing the transformation from TiO_2_ to TiCl_4_. In comparison, Zhang et al. [39] reported that TiCl_4_ was generated at 140.7 ps in the NaCl–C–Cl_2_–TiO_2_ system. This clearly demonstrates that the molten salt carbothermic chlorination rate is significantly accelerated with the involvement of Fe. This process is consistent with the conclusions from the earlier AIMD calculations in this study, which indicated that iron enhances the activity of neighboring carbon by promoting electron transfer, thereby facilitating the reduction and chlorination of titanium oxides.

#### 3.3.2. Effect of Temperature on the Interaction of Fe with System Components

**Figure 11:**
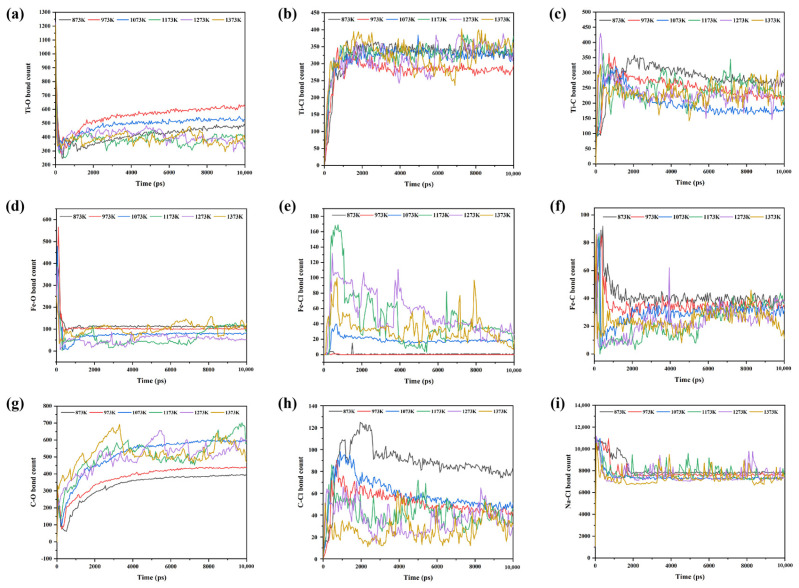
Time-dependent variation in the number of bonds formed between specific atomic pairs at different temperatures in the NaCl–C–Cl_2_–FeTiO_3_ system: (**a**) Ti-O; (**b**) Ti-Cl; (**c**) Ti-C; (**d**) Fe-O; (**e**) Fe-Cl; (**f**) Fe-C; (**g**) C-O; (**h**) C-Cl; (**i**) Na-Cl.

To further analyze the effect of Fe on the components of the titanium oxide molten salt carbothermic chlorination process at different temperatures and to gain a deeper understanding of the catalytic mechanism of iron, the number of bonds formed between specific atoms in the NaCl–C–Cl_2_–FeTiO_3_ system was statistically tracked over time at temperature intervals of 100 K within the 873 K~1373 K range. The results are shown in Figure 11.

Figure 11 shows the variation in the number of bonds formed between different atomic pairs over simulation time during the molten salt carbothermic chlorination process of titanium oxides containing Fe at different temperatures. As seen in Figure 11a,d,h, the number of Ti-O, Fe-O, and C-Cl bonds generally decreases with increasing temperature, indicating that higher temperatures facilitate the reduction of titanium oxides and iron oxides. It also suggests that at high temperatures, carbon-chlorine bonds are more easily dissociated, promoting the release of chlorine. Therefore, a noticeable fluctuation in the number of C-Cl bonds is observed within the temperature range of 1173 K~1373 K. As shown in Figure 11e, the number of Fe-Cl bonds increases with temperature, suggesting that higher temperatures accelerate the formation of iron chloride products. In Figure 11b,g,i, the number of Ti-Cl, C-O, and Na-Cl bonds generally increases with temperature from 873 K to 1073 K, indicating that the increase in temperature within this range not only promotes reduction but also accelerates the formation of chlorination products. However, in the 1173 K~1373 K range, the number of Ti-Cl, C-O, and Na-Cl bonds fluctuates significantly with increasing temperature, and the overall number is similar to that at 1073 K, suggesting that further temperature increase does not significantly enhance the reduction and chlorination efficiency. As seen in Figure 11c,f, the number of Ti-C and Fe-C bonds decreases with increasing temperature in the 873 K~1073 K range, and fluctuates significantly in the 1173 K~1373 K range, although the overall number is similar to that at 1073 K. This indicates that further temperature increase in this range does not strengthen the Ti-C and Fe-C interactions. Combined with the decrease in the number of C-Cl bonds in this temperature range, and the increase in iron chlorination reactions, it can be concluded that further temperature rise will promote the formation of iron chlorides and weaken the bonding between iron and carbon, which is detrimental to the catalytic effect of iron. Therefore, it can be preliminarily concluded that around 1073 K, iron exhibits a higher catalytic effect in the molten salt carbothermic chlorination of titanium oxides in sodium chloride.

**Figure 12:**
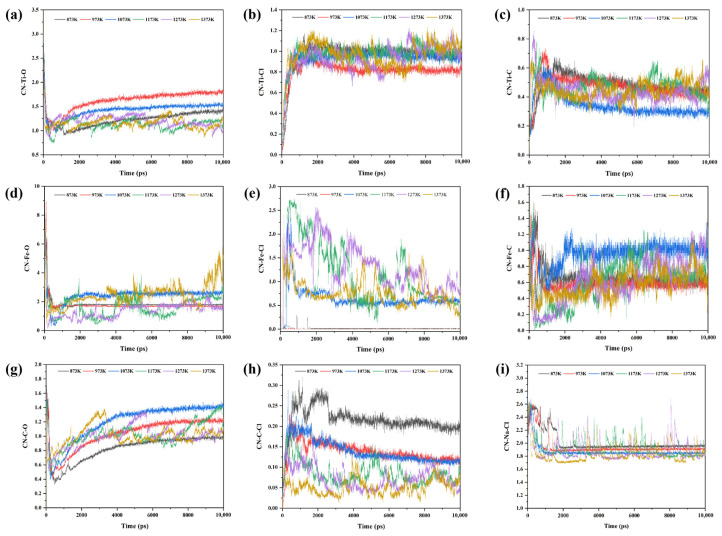
Time-dependent variation in the coordination numbers of specific atomic pairs at different temperatures in the NaCl–C–Cl_2_–FeTiO_3_ system: (**a**) Ti-O; (**b**) Ti-Cl; (**c**) Ti-C; (**d**) Fe-O; (**e**) Fe-Cl; (**f**) Fe-C; (**g**) C-O; (**h**) C-Cl; (**i**) Na-Cl.

Further analysis was conducted on the variation in coordination numbers of the above atomic pairs over time, and the results are shown in Figure 12. A comparison of Figure 11 and Figure 12 reveals that the trends in coordination numbers over time for Ti-O, Ti-Cl, Ti-C, C-Cl, and Na-Cl are generally consistent with the trends in the number of bonds over time. However, the coordination numbers over time for Fe-C, Fe-O, Fe-Cl, and C-O show different trends compared to the bond lengths over time. Therefore, the focus is placed on discussing the variation in coordination numbers for Fe-C, Fe-O, Fe-Cl, and C-O over time. As shown in Figure 12f, in the variation in coordination numbers over time for Fe and C at different temperatures, although the coordination number of Fe-C generally increases with temperature, it is notably higher at 1073 K compared to other temperatures. Combining this with the previous analysis of Figure 11f, it can be concluded that the Fe-C bonding is more stable at 1073 K, which has a significant catalytic effect on the reduction process. As shown in Figure 12d,e, the coordination number of Fe-O generally decreases with increasing temperature, while the coordination number of Fe-Cl increases with temperature. It is worth noting that in the high-temperature range of 1173 K~1373 K, the coordination numbers of Fe-O and Fe-Cl exhibit large fluctuations over time. However, compared to the lower temperatures, the coordination number of Fe-O is lower and Fe-Cl is higher. By the end of the simulation, the coordination numbers in this temperature range are close to those at 1073 K, indicating that high temperatures can promote the reduction of iron oxides and the formation of iron chlorides. However, effective reduction and chlorination can already be achieved near 1073 K. As shown in Figure 12g, the coordination number of C-O increases overall with the simulation time from 873 K to 1373 K. Within the range of 873 K~1073 K, the coordination number of C-O shows a noticeable increase with temperature, while in the 1173 K~1373 K range, the coordination number of C-O fluctuates significantly. Notably, the coordination number of C-O at 1073 K is overall higher than at other temperatures. Combining this with the highest coordination number of Fe-C at 1073 K, it can be concluded that at this temperature, the interaction between C and O, enhanced by Fe, is stronger compared to other temperatures, further confirming that Fe exhibits excellent catalytic effects around 1073 K.

**Figure 13:**
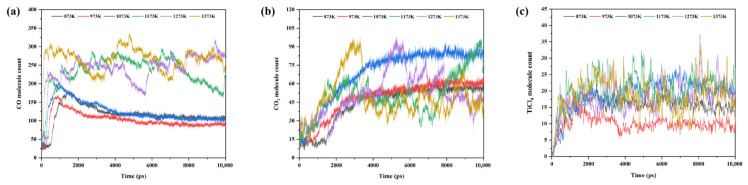
Time-dependent variation in the number of specific molecules in the NaCl–C–Cl_2_–FeTiO_3_ system: (**a**) CO; (**b**) CO_2_; (**c**) TiCl_4_.

To further elucidate the effect of Fe on the reduction and chlorination of titanium oxides at different temperatures, the numbers of CO, CO_2_, and TiCl_4_ molecules in the NaCl–C–Cl_2_–FeTiO_3_ system were statistically analyzed as a function of time over the temperature range of 873 K~1373 K, as shown in Figure 13. As can be seen from Figure 13a, the amount of CO generated generally increases with increasing temperature, and the number of CO molecules in the 1173 K~1373 K range is significantly higher than that in the 873 K~1073 K range, indicating that an increase in temperature is favorable for CO generation. In addition, it is worth noting that within the 873 K~1073 K range, the number of CO molecules reaches its peak at around 500 ps, then gradually decreases due to participation in subsequent reduction reactions, and becomes stable at around 4000 ps. In contrast, within the 1173 K~1373 K range, the number of CO molecules exhibits large fluctuations after reaching the peak value, indicating that although high temperature promotes CO formation to a certain extent, excessively high temperatures also cause some CO molecular structures to exist in a metastable state. As shown in Figure 13b, before 4000 ps, the number of CO_2_ molecules generally increases with increasing temperature. However, after 4000 ps, the number of CO_2_ molecules in the 1173 K~1373 K range shows greater fluctuations than that in the 873 K~1073 K range, indicating that, similar to CO, CO_2_ is also in an unstable state at high temperatures. Notably, at 1073 K, the number of CO_2_ molecules gradually becomes higher than that at other temperatures with simulation time, and the fluctuation of the curve is relatively small, indicating that a relatively large amount of CO_2_ can be continuously and stably generated near this temperature. In addition, the number of CO_2_ molecules increases sharply from 500 ps and gradually stabilizes at around 4000 ps. Combined with Figure 13a, this suggests that CO_2_ is mainly formed by the further conversion of CO. However, judging from the numerical changes of the two species, the consumed CO is not completely converted into CO_2_, indicating that carbon oxides may also interact with other components in the system during the conversion process, thereby intensifying the dynamic fluctuations in molecular numbers, as described in the previous thermodynamic analysis.

As shown in Figure 13c, the number of TiCl_4_ molecules formed exhibits considerable fluctuations at all temperatures. However, the amount of TiCl_4_ generated in the 1073~1373 K range is higher than that in the 873~973 K range, indicating that an increase in temperature promotes the formation of TiCl_4_ to a certain extent. Notably, the overall amount of TiCl_4_ formed at 1073 K remains at a relatively high level, suggesting that efficient conversion from TiO_2_ to TiCl_4_ can be achieved when the temperature is raised to around this value, which is consistent with the result shown in Figure 3. In summary, although increasing temperature can promote the formation of TiCl_4_ to some extent, the catalytic effect of Fe is no longer significantly enhanced at excessively high temperatures. Around 1073 K, both the yield and stability of the products can reach relatively favorable levels.

**Figure 14:**
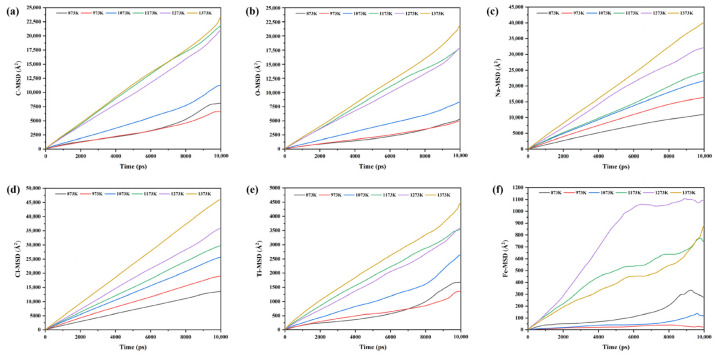
Mean square displacements of various components in the NaCl–C–Cl_2_–FeTiO_3_ system at different temperatures: (**a**) C; (**b**) O; (**c**) Na; (**d**) Cl; (**e**) Ti; (**f**) Fe.

**Figure 15:**
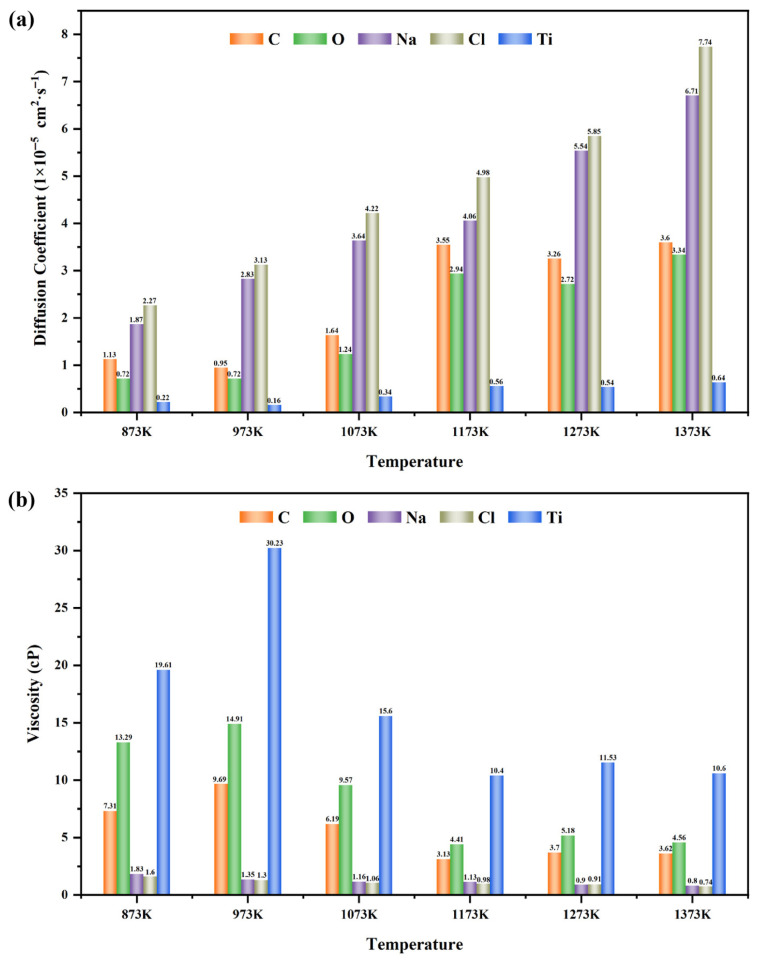
Diffusion coefficients and viscosities of various components in the NaCl–C–Cl_2_–FeTiO_3_ system: (**a**) Diffusion Coefficient; (**b**) Viscosity.

To further analyze the effect of Fe on the migration behavior of various components in the NaCl–C–Cl_2_–FeTiO_3_ system at different temperatures, the temperature-dependent variations in the mean square displacement, diffusion coefficient, and viscosity of each component in the system were discussed, as shown in Figure 14 and Figure 15.

As shown in Figure 14, the mean square displacements of all components in the system generally increase with increasing temperature, indicating that higher temperatures are favorable for the diffusion and migration of the components, thereby making effective collisions and interactions more likely during thermal motion. In addition, as shown in Figure 14a,b,f, C, O, and Fe are more strongly affected by temperature, and their mean square displacements in the high-temperature range of 1173~1373 K are significantly greater than those in the 873~1073 K range. This suggests that the diffusion of Fe is markedly accelerated at high temperatures, greatly increasing its probability of contacting C. As a result, the Fe-activated C can reduce titanium oxides more efficiently, thereby accelerating the formation of carbon oxide products mainly composed of CO and CO_2_.

To quantitatively characterize the transport properties of each component in the molten salt environment at different temperatures, further analysis was carried out on the diffusion coefficients and viscosities. Considering that the proportion of Fe in the NaCl–C–Cl_2_–FeTiO_3_ system is relatively low (only 63 out of 10,973 atoms), and to avoid introducing large errors, the quantitative analysis was focused on the major components, including C, O, Na, Cl, and Ti. The results are shown in Figure 15. As shown in Figure 15a,b, with increasing temperature, the diffusion coefficients of all components increase to different extents, whereas the viscosity exhibits a clear negative correlation with the diffusion coefficient. As shown in Figure 15a, at 1073 K, the diffusion coefficients of C, O, Na, Cl, and Ti are 1.64 × 10^−5^ cm^2^·s^−1^, 1.24 × 10^−5^ cm^2^·s^−1^, 3.64 × 10^−5^ cm^2^·s^−1^, 4.22 × 10^−5^ cm^2^·s^−1^, and 0.34 × 10^−5^ cm^2^·s^−1^, respectively. As shown in Figure 15b, the viscosity gradually decreases with increasing diffusion coefficient. At 1073 K, the viscosities of C, O, Na, Cl, and Ti are 6.19 cP, 9.57 cP, 1.16 cP, 1.06 cP, and 15.6 cP, respectively. A comparison of the transport parameters of Na and Cl with previous studies shows good agreement. Lu et al. [57] reported that at 1100 K, the diffusion coefficients of Na and Cl in molten salt were 9 × 10^−5^ cm^2^·s^−1^ and 7 × 10^−5^ cm^2^·s^−1^, respectively. Tasidou et al. [58] pointed out that the viscosity of molten NaCl at 1090 K is approximately 1.01 cP, both of which are basically consistent with the parameters calculated in this study. In summary, as the temperature increases, Fe can strengthen its catalytic effect by increasing its displacement, thereby accelerating the migration of the components, promoting the reaction process, and reducing the viscosities of the various components in the molten salt.

## 4. Conclusions

This study systematically revealed the behavioral characteristics and catalytic mechanism of Fe during the molten salt carbothermic chlorination of titanium oxides by integrating thermodynamic calculations, ab initio molecular dynamics (AIMD) simulations, and deep potential molecular dynamics (DPMD) simulations in a multiscale framework. The results indicate that Fe can catalytically promote both the reduction and chlorination processes by regulating electron transfer and enhancing the activity of C in the system. This study elucidates the catalytic mechanism of Fe at the atomic scale and provides theoretical support for temperature optimization and capacity improvement in the molten salt chlorination process of titanium oxides.

(1) The thermodynamic results show that, in addition to the formation of major products such as TiCl_4_, CO, and CO_2_, the molten salt chlorination process is also accompanied by the presence of intermediate products represented by C_3_O_2_, NaFeCl_4_, Ti_2_Cl_4_O_2_, and COCl. These intermediates reflect the transient characteristics of the interactions among the various components and provide a theoretical basis for the subsequent molecular dynamics analysis.

(2) The AIMD results demonstrate that Fe can significantly enhance the activity of C and improve its electron migration capability through coordination with carbon. In the presence of Fe, the activated carbon can efficiently weaken the Ti-O bond and promote the formation of the C-O bond, causing the cleavage of the Ti-O bond to occur markedly earlier than in the absence of Fe, thereby greatly accelerating the reduction of titanium oxides. In addition, Fe can directly transfer electrons to C or transfer electrons to Ti through bridged carbon, thereby facilitating C-Cl bond dissociation and Ti-Cl bond formation, and thus achieving the synergistic catalysis of both the reduction and chlorination processes.

(3) The DPMD simulations indicate that Fe exerts a significant influence on the carbon cluster structure and the migration behavior of the various components in the system. The presence of Fe promotes the dissociation and diffusion of carbon clusters, while also strengthening the interaction between activated carbon and titanium oxides, thereby accelerating the reduction process and significantly shortening the formation time of TiCl_4_. In addition, analysis of the catalytic effect of Fe at different temperatures shows that the coordination numbers of Fe-C and C-O reach relatively high levels at 1073 K. Around this temperature, the formation efficiency of TiCl_4_ is high and the product state remains relatively stable. Although further increasing the temperature can enhance diffusion, the Fe-C interaction becomes weaker, and the catalytic effect is no longer significantly improved.

## Figures and Tables

**Table 1:** Equilibrium components and their contents in the NaCl–C–Cl_2_–FeTiO_3_ system at 1073 K.

Balancing Components						
Element	TiO_2_	Ti(g)	TiCl(g)	TiCl_2_	TiCl_2_(g)	TiCl_3_
Concentration(kmol)	3.6 × 10^−10^	2.1 × 10^−42^	7.7 × 10^−30^	1.3 × 10^−11^	3.1 × 10^−15^	1 × 10^−4^
Element	TiCl_3_(g)	TiCl_4_(l)	TiCl_4_(g)	TiC	TiO	TiClO
Concentration(kmol)	7.7 × 10^−5^	19.09	330.9	4.1 × 10^−20^	1.6 × 10^−15^	1.1 × 10^−8^
Element	TiClO(g)	TiCl_2_O(g)	TiCl_2_O_2_(g)	TiCl_3_O(g)	TiCl_3_O_2_(g)	Ti_2_Cl_4_O_2_(g)
Concentration(kmol)	9.5 × 10^−22^	6.1 × 10^−10^	2.3 × 10^−29^	1 × 10^−14^	2 × 10^−22^	1.1 × 10^−21^
Element	FeO(l)	FeO	FeO_2_(g)	FeOCl(g)	Fe_2(_g)	Fe_2_C
Concentration(kmol)	6.4 × 10^−9^	1.5 × 10^−8^	9.1 × 10^−30^	7.7 × 10^−17^	4.3 × 10^−45^	1.9 × 10^−20^
Element	Fe_3_C	FeCO_3_	FeTi	Fe(g)	FeCl(g)	FeCl_2_
Concentration(kmol)	1.8 × 10^−32^	2.3 × 10^−15^	4.2 × 10^−37^	1.2 × 10^−20^	6 × 10^−15^	91.92
Element	FeCl_2_(g)	FeCl_3_	FeCl_3_(g)			
Concentration(kmol)	7.74	37.15	204.34			
Element	C	CO(g)	CO_2_(g)	COCl(g)	COCl_2_(g)	C_2_(g)
Concentration(kmol)	339.41	669.93	189.44	0.005	1.17	5.6 × 10^−30^
Element	C_3_(g)	C_2_O(g)	C_3_O_2_(g)	C(g)	CCl(g)	CCl_2_(g)
Concentration(kmol)	2.9 × 10^−30^	3.7 × 10^−17^	4.8 × 10^−13^	5.6 × 10^−25^	1.7 × 10^−17^	1.9 × 10^−8^
Element	CCl_3_(g)	CCl_4_(g)	C_2_Cl(g)	C_2_Cl_2_(g)	C_2_Cl_3_(g)	C_3_Cl_3_(g)
Concentration(kmol)	2.1 × 10^−5^	0.01	1.8 × 10^−21^	4.1 × 10^−9^	4.8 × 10^−11^	1.7 × 10^−16^
Element	NaCl	NaCl(l)	NaCl(g)	Na(g)	NaO(g)	Na_2_O(g)
Concentration(kmol)	2467	2531.6	0.96	1.7 × 10^−12^	1.2 × 10^−23^	7.3 × 10^−33^
Element	Na_2_Cl_2_(g)	Na_3_Cl_3_(g)	NaFeCl_4_(g)			
Concentration(kmol)	0.12	6 × 10^−4^	0.05			
Element	Cl(g)	Cl_2_(g)	Cl_3_(g)	Cl_4_(g)	O(g)	
Concentration(kmol)	2.65	822.3	4.6 × 10^−16^	2.5 × 10^−17^	1.5 × 10^−15^	

## Data Availability

The original contributions presented in this study are included in the article/Supplementary Materials. Further inquiries can be directed to the corresponding author.

## Supplementary Materials

The following supporting information can be downloaded at: https://www.mdpi.com/article/10.3390/ma19091746/s1. Table S1: Bond length information for specific atom pairs in AIMD; Table S2: Population data information for specific atom pairs in AIMD; Table S3: Mulliken charge information for specific atom pairs in AIMD.
