# Supplementary material for: Iron-Catalyzed Chlorination of Titanium Oxides in Molten Salts: A Deep Neural Network-Based Mechanistic Study
Source: Materials (Basel). 2026 Apr 24;19(9):1746. doi: 10.3390/ma19091746 (PMC13165055; doi:10.3390/ma19091746)
Supplement: Supplementary file 1 [file materials-19-01746-s001.zip › materials-4223981-supplementary.pdf]

Supplementary Materials: Iron-Catalyzed Chlorination of Titanium Oxides in Molten Salts: A Deep Neural Network-Based Mechanistic Study

Authors: Liangliang Gu, Jie Zhou, Wei Liu, Yuanyuan Chen, Linfei Li, Ronggang Sun, Rong Yu, Xiumin Chen, Yunmin Chen

Section S1. Electronic property data in AIMD results

1.1. Bond Lengths

Table S1. Bond length information for specific atom pairs in AIMD

Bond	0 ps	0.143 ps	0.24 ps	0.303 ps	0.35 ps	0.49 ps	0.874 ps	1.131 ps	2.5 ps
Ti 49-O 18	1.99	1.84	2.02	1.94	1.83	2.02	1.82	1.99	1.87
Ti 49-O 56	2.05	——	——	——	——	——	——	——	——
Ti 49-Cl 141	——	——	2.45	2.29	2.36	2.38	2.44	2.33	2.13
Ti 49-Cl 163	2.24	2.4	2.29	2.29	2.2	2.2	2.43	2.24	2.41
Ti 49-Cl 164	2.54	2.59	2.75	——	——	——	——	——	——
Cl 164-Ti 50	2.42	2.36	2.48	——	——	——	——	——	——
Ti 49-C 56	1.93	2.01	1.97	1.96	2.07	1.87	1.87	1.83	1.86
C 56-Fe 3	1.83	1.69	1.79	1.69	1.92	1.82	1.81	1.84	1.76
Fe 3-O 56	1.94	1.85	1.88	2.04	1.97	1.88	1.93	1.87	2.23
O 56-C 91	1.37	1.34	1.35	1.26	1.35	1.34	1.25	1.35	1.22
C 91-Fe 2	1.77	1.88	1.75	1.89	1.75	1.84	1.74	1.82	1.75
Ti 15-O 46	2.01	2.38	2.29	2.03	2.06	2.04	1.89	——	——
O 46-Ti 41	1.99	1.97	2.42	2.05	2.75	——	2.73	1.95	2.08
O 46-C 85	1.43	1.28	1.26	1.35	1.43	1.26	1.31	1.31	1.26
C 85-Fe 3	2.02	2.05	1.87	1.97	1.85	1.85	1.9	1.81	1.76
Cl 141-C 56	1.69	1.72	1.93	2.25	——	——	——	——	——
Cl 141-Na 27	3.77	2.61	2.87	2.53	2.67	3.9	——	——	——
Cl 141-Na 57	4.05	3.06	3.3	3.86	4.05	3.63	3.84	——	3.04

1.2. Population Data

Table S2. Population data information for specific atom pairs in AIMD

Bond	0 ps	0.143 ps	0.24 ps	0.303 ps	0.35 ps	0.49 ps	0.874 ps	1.131 ps	2.5 ps
Ti 49-O 18	0.3	0.39	0.32	0.29	0.39	0.31	0.42	0.34	0.39
Ti 49-O 56	0.15	——	——	——	——	——	——	——	——
Ti 49-Cl 141	——	——	0.15	0.37	0.36	0.56	0.49	0.44	0.49
Ti 49-Cl 163	0.61	0.61	0.65	0.67	0.66	0.66	0.47	0.57	0.57
Ti 49-Cl 164	0.32	0.3	0.23	——	——	——	——	——	——
Cl 164-Ti 50	0.4	0.37	0.41	——	——	——	——	——	——
Ti 49-C 56	0.46	0.41	0.46	0.54	0.56	0.72	0.73	0.73	0.76
C 56-Fe 3	0.59	0.84	0.74	0.88	0.52	0.54	0.57	0.34	0.64
Fe 3-O 56	0.24	0.26	0.24	0.21	0.29	0.33	0.31	0.29	0.1
O 56-C 91	0.67	0.79	0.73	0.84	0.75	0.75	0.88	0.73	0.97
C 91-Fe 2	0.67	0.65	0.5	0.55	0.45	0.41	0.59	0.45	0.41
Ti 15-O 46	0.32	0.22	0.3	0.27	0.38	0.36	0.28	——	——
O 46-Ti 41	0.21	0.2	0.16	0.23	0.1	——	0.06	0.31	0.27
O 46-C 85	0.52	0.77	0.86	0.68	0.64	0.84	0.78	0.75	0.84
C 85-Fe 3	0.39	0.37	0.26	0.37	0.41	0.38	0.45	0.49	0.23
Cl 141-C 56	0.56	0.41	0.22	0.04	——	——	——	——	——
Cl 141-Na 27	——	0.07	0	0.05	0.1	——	——	——	——
Cl 141-Na 57	——	——	——	——	——	——	——	——	——

1.3. Mulliken charge

Table S3. Mulliken charge information for specific atom pairs in AIMD

Atom	0 ps	0.143 ps	0.24 ps	0.303 ps	0.35 ps	0.49 ps	0.874 ps	1.131 ps	2.5 ps
**Fe 2**	**0.9**	**0.9**	**1.05**	**0.95**	**1.13**	**1.02**	**0.77**	**1.03**	**1.06**
**Fe 3**	**0.56**	**0.42**	**0.58**	**0.37**	**0.57**	**0.67**	**0.69**	**0.69**	**0.66**
**Ti 15**	**1.37**	**1.33**	**1.27**	**1.39**	**1.31**	**1.25**	**1.31**	**1.23**	**1.16**
**Ti 41**	**1.17**	**0.97**	**0.93**	**0.93**	**0.76**	**0.83**	**0.81**	**0.8**	**0.74**
**Ti 49**	**1.06**	**1**	**0.89**	**0.8**	**0.86**	**0.77**	**0.87**	**0.82**	**0.76**
**Ti 50**	**0.89**	**0.94**	**1**	**1.11**	——	——	——	——	——
**C 56**	**-0.59**	**-0.63**	**-0.64**	**-0.47**	**-0.6**	**-0.63**	**-0.66**	**-0.58**	**-0.59**
**C 85**	**-0.22**	**-0.07**	**-0.05**	**-0.05**	**-0.06**	**-0.01**	**0.04**	**0**	**-0.04**
**C 86**	**-0.1**	**-0.3**	**-0.23**	**-0.2**	**-0.24**	**-0.27**	**-0.23**	**-0.2**	**-0.27**
**C 91**	**0.21**	**0.19**	**0.17**	**0.29**	**0.21**	**0.27**	**0.37**	**0.24**	**0.26**
**O 18**	**-0.73**	**-0.66**	**-0.66**	**-0.65**	**-0.64**	**-0.68**	**-0.67**	**-0.67**	**-0.65**
**O 46**	**-0.57**	**-0.53**	**-0.55**	**-0.56**	**-0.55**	**-0.53**	**-0.53**	**-0.52**	**-0.51**
**O 47**	**-0.61**	**-0.64**	**-0.64**	**-0.62**	**-0.62**	**-0.63**	**-0.7**	**-0.66**	**-0.66**
**O 56**	**-0.55**	**-0.46**	**-0.49**	**-0.51**	**-0.5**	**-0.52**	**-0.48**	**-0.51**	**-0.51**
**Cl 141**	**0.04**	**0**	**-0.05**	**-0.28**	**-0.35**	**-0.38**	**-0.42**	**-0.45**	**-0.38**
**Cl 163**	**-0.3**	**-0.33**	**-0.27**	**-0.29**	**-0.26**	**-0.3**	**-0.47**	**-0.28**	**-0.4**
**Cl 164**	**-0.33**	**-0.31**	**-0.35**	**-0.33**	——	——	——	——	——
**Na 27**	**0.62**	**0.78**	**0.79**	**0.79**	**0.77**	**0.77**	**0.7**	**0.58**	**0.5**
**Na 57**	**0.59**	**0.66**	**0.7**	**0.67**	**0.63**	**0.55**	**0.59**	**0.59**	**0.67**
